# Regulation of IGFBP-2 expression during fasting

**DOI:** 10.1042/BJ20141248

**Published:** 2015-04-17

**Authors:** Hye Suk Kang, Mi-Young Kim, Seung-Jae Kim, Jae-Ho Lee, Yong-Deuk Kim, Young-Kyo Seo, Jae-Hoon Bae, Goo-Taeg Oh, Dae-Kyu Song, Yong-Ho Ahn, Seung-Soon Im

**Affiliations:** *Department of Physiology, Keimyung University School of Medicine, Daegu 704-701, Republic of Korea; †Department of Biochemistry and Molecular Biology, Yonsei University College of Medicine, Seoul 120-752, Republic of Korea; ‡School of Life Sciences, UNIST, Ulsan 689-798, Republic of Korea; §Division of Life and Pharmaceutical Sciences, Ewha Womans University, Seoul 120-750, Republic of Korea

**Keywords:** fasting, gene expression, insulin-like growth factor-1 (IGF-1), insulin-like growth factor-binding protein 2 (IGFBP-2), liver, peroxisome-proliferator-activated receptor α (PPARα), G6pc, glucose-6-phosphatase catalytic subunit, HEK, human embryonic kidney, IGF, insulin-like growth factor, IGF-1R, insulin-like growth factor-1 receptor, IGFBP, insulin-like growth factor-binding protein, mTOR, mammalian target of rapamycin, Pck1, phosphoenolpyruvate carboxykinase 1, PPAR, peroxisome-proliferator-activated receptor, PPRE, peroxisome-proliferator-responsive element, qPCR, quantitative PCR, S6K, S6 kinase, WT, wild-type

## Abstract

Insulin-like growth factor (IGF)-binding protein-2 (IGFBP-2), one of the most abundant circulating IGFBPs, is known to attenuate the biological action of IGF-1. Although the effect of IGFBP-2 in preventing metabolic disorders is well known, its regulatory mechanism remains unclear. In the present study, we demonstrated the transcriptional regulation of the *Igfbp-2* gene by peroxisome-proliferator-activated receptor (PPAR) α in the liver. During fasting, both *Igfbp-2* and *PPARα* expression levels were increased. Wy14643, a selective PPARα agonist, significantly induced *Igfbp-2* gene expression in primary cultured hepatocytes. However, *Igfbp-2* gene expression in *Pparα* null mice was not affected by fasting or Wy14643. In addition, through transient transfection and chromatin immunoprecipitation assay in fasted livers, we determined that PPARα bound to the putative PPAR-responsive element between −511 bp and −499 bp on the *Igfbp-2* gene promoter, indicating that the *Igfbp-2* gene transcription is activated directly by PPARα. To explore the role of PPARα in IGF-1 signalling, we treated primary cultured hepatocytes with Wy14643 and observed a decrease in the number of IGF-1 receptors (IGF-1Rs) and in Akt phosphorylation. No inhibition was observed in the hepatocytes isolated from *Pparα* null mice. These results suggest that PPARα controls IGF-1 signalling through the up-regulation of hepatic *Igfbp-2* transcription during fasting and Wy14643 treatment.

## Abstract 2

Insulin-like growth factor (IGF)-binding protein-2 (IGFBP-2) is known to attenuate the biological action of IGF-1, but its regulatory mechanism remains unclear. We demonstrate the transcriptional regulation of the hepatic *Igfbp-2* gene by peroxisome-proliferator-activated receptor (PPAR) α during fasting. We also show how PPARα controls IGF-1 signalling through IGFBP-2.

## INTRODUCTION

The insulin-like growth factor (IGF)-binding protein (IGFBP) is known as a carrier protein for IGF-1 [1]. There are currently seven characterized IGFBPs: IGFBP-1 to IGFBP [1]. IGFBPs also function as modulators of IGF bioactivity and availability [2]. The binding affinity of IGFBPs for IGFs is higher than that of type I IGF receptors. The biological function of IGFBPs was identified when it was demonstrated that IGFBPs are capable of important biological roles that are independent of their ability to bind to IGFs [3]. IGFBP-2, an abundantly secreted protein [4], is expressed in the adult liver, adipocytes and central nervous system, and plays a key role in the regulation of metabolic homoeostasis and insulin sensitivity [5,6].

IGF-1 not only plays a key role in regulating growth and development, but also has a variety of pathophysiological functions related to cardiovascular disease, cancer, diabetes, liver cirrhosis and Laron syndrome [7,8]. It is a single-chain polypeptide hormone with endocrine, paracrine and autocrine properties and is produced mainly in the liver. However, its synthesis has also been shown to occur in a variety of other tissues, such as the kidney, pancreas, skin and testes [7,9]. Because IGF-1 shares structural homology with insulin and interacts with the same membrane receptors [10], its key pathway is regulated by the phosphoinositide-3-kinase (PI3K)–Akt–mTOR (mammalian target of rapamycin), mitogen-activated protein kinase (MAPK)–Ras–ERK (extracellular-signal-regulated kinase) and the Janus kinase (JAK)–signal transducer and activator of transcription (STAT) pathway [11]. Importantly, the bioactivity of IGF-1 is effectively altered by high-affinity binding to IGFBP, which subsequently attenuates IGF-1 activity [11,12]. At least six IGFBPs are known to be involved in the expression and biological activities of IGF-1 by binding to the IGF-1 receptor (IGF-1R) [13]. IGF-1R consists of a α_2_β_2_ heterotetrameric complex with extracellular α-subunits containing ligand-binding sites. Each α-subunit of IGF-1R binds to one of two membrane-spanning β-subunits with tyrosine kinase domains [14].

Peroxisome-proliferator activated receptor (PPAR)α is a member of the superfamily of nuclear receptors that function as transcription factors involved in the regulation of a variety of metabolic processes, such as inflammation, insulin sensitivity and glucose and lipid metabolism [15]. PPARα is highly expressed in the liver and adipose tissue and is present in a diverse set of tissues including the heart, kidney and intestines [15,16]. PPARα is stimulated by ligands such as Wy14643 in addition to fenofibrate and the fasting state, elevating fatty acid oxidation, ketogenesis, bile acid synthesis and gluconeogenesis, as well as improving inflammation and insulin sensitivity in response [16,17]. Previous reports have shown that PPARα promotes apoptosis and inhibits cellular functions by IGF-1R signalling and Akt phosphorylation in various cancer cells [18–20]. Although PPARα is associated with an IGF-1-dependent pathway *in vitro*, any potential link between PPARα and IGF-1 signalling has yet to be determined in an *in vivo* system.

In the present study, we identified PPARα as a key regulator of *Igfbp-2* gene transcription in the fasting state and revealed up-regulation of IGFBP-2 by Wy14643 exposure. IGF-1 sensitivity was prevented via down-regulation of the IGF-1-dependent pathway under fasting *in vivo* and *in vitro* conditions. These results suggest that regulation of the IGFBP-2–IGF-1 network by a PPARα agonist may provide a novel molecular mechanism for improving physiological changes via controlling IGF-1 bioactivity.

## EXPERIMENTAL

### Materials

Wy14643 (Sigma–Aldrich) and recombinant human IGF-1 (Life Technologies) were dissolved in the recommended solvents. Antibodies against p-IGF-1R, p-Akt, Akt, p-mTOR and p-S6 kinase (S6K) were purchased from Cell Signaling Technology and anti-PPARα, IGFBP-2, IGF-1R and β-actin were from Santa Cruz Biotechnology.

### Experimental animals

Male C57BL6 mice (Jung-Ang Experimental Animals, Seoul, Republic of Korea) and *Pparα* null mice at 8-weeks-old were used in the experiments, as described previously [21]. For fasting and feeding experiments, mice were fed or fasted for 24 h.

All animal experiments were performed in accordance with the rules and regulations of the Institutional Animal Use and Care Committee (IAUCC), Keimyung University School of Medicine.

### Isolation and culture of primary mouse hepatocytes

Mouse primary hepatocytes were isolated from wild-type (WT) and *Pparα* null mice. The hepatocytes were used for quantitative PCR (qPCR) and immunoblot analyses. The hepatocyte isolation method used was described previously [22].

### qPCR analysis

Total RNA was isolated from mouse primary hepatocytes and livers using the TRIzol method (Invitrogen). cDNA was synthesized using a SuperScript III First-Strand cDNA Synthesis kit (Invitrogen) and used for qPCR with LightCycler Real-Time PCR systems (Roche Applied Science). All data were normalized to ribosomal L32 expression. The following primer sets were used: *Pparα*: forward, 5′-AGAGCCCCATCTGTCCTCTC-3′; reverse, 5′-ACTGGTAG-TCTGCAAAACCAAA-3′; *Igfbp-1*: forward, 5′-ATCAGC-CCATCCTGTGGAAC-3′; reverse, 5′-TGCAGCTAATCTCTC TAGCACTT-3′; *Igfbp-2*: forward, 5′-CAGACGCTACGCTG-CTATCC-3′; reverse, 5′-CTCCCTCAGAGTGGTCGTCA-3′; *Pck1* (phosphoenolpyruvate carboxykinase 1): forward, 5′-CCACAGCTGCTGCAGAACA-3′; reverse, 5′-GAAGGGTCG-CATGGCAAA-3′; *L32*: forward, 5′-ACATTTGCCCTGAA-TGTGGT-3′; reverse, 5′-ATCCTCTTGCCCTGATCCTT-3′; human *Igfbp-2*: forward, 5′-GACAATGGCGATGACCACTCA-3′; reverse, 5′-CAGCTCCTTCATACCCGACTT-3′; human *ACTB* (β-actin): forward, 5′-GGCATCCTCACCCTGAAGTA-3′.

### Immunoblotting

Mouse primary hepatocytes were isolated and processed according to a method described previously [21]. The membranes were probed with the indicated antibodies and then developed using an enhanced chemiluminescent Western blot detection kit (GE Healthcare). The intensities of the bands were calculated using ImageJ software (NIH), verifying non-saturation and subtracting the background. Values are expressed as the integrals (target area density) of each band (normalized to total indicated protein band).

### ChIP assay

The ChIP assay was performed as described previously [21]. Briefly, mice were fasted for 24 h and refed for 12 h. Livers were then collected from the mice and fixed with paraformaldehyde for 5 min prior to performing the ChIP assay using anti-PPARα. Liver tissue (25 mg) was used for each ChIP/antibody sample. The final DNA extractions were quantified by PCR with primers for the putative peroxisome-proliferator-responsive element (PPRE; −500/−300) region of the *Igfbp-2* promoter. PPRE on the glucose-6-phosphatase catalytic subunit (*G6pc*) promoter was used as a positive control for the ChIP experiment with an anti-PPARα antibody. Raw *C*_T_ values obtained from the ChIP samples were divided by *C*_T_ values obtained from the appropriate input samples to analyse the percentage input values. The specific primers used for PCR were as follows: mouse *Igfbp-2c*, forward 5′-TCATTACTTGCAGCGGTGAGC-3′ and reverse 5′-CCGGGGAAACACAAAAGCAG-3′; mouse *G6pc-Ppre*, forward 5′-GCTGTTTTTGTGTGCCTGTT-3′ and reverse 5′-TGCTATCAGTCTGTGCCTTGG-3′; mouse *Gapdh*, forward 5′-CCTGGAGAAACCTGCCAAGTA-3′ and reverse 5′-TGGAAGAGTGGGAGTTGCTGT-3′.

### ELISA for IGFBP-2

Serum was obtained from mice and primary hepatocytes. Secreted IGFBP-2 was quantified using mouse serum and the Quantikine mouse IGFBP-2 kit (Raybiotech). Serum (50 μl) was diluted with 50 μl of dilute buffer. Then, 80 μl of 12.5% 1 M formic acid and 87.5% ethanol mixture were added and incubated for 30 min at room temperature to free IGFBP-2. The prepared samples were used in each well of the Quantikine kit, as instructed in the manual. Conditioned medium was assayed for secreted IGFBP-2 using the IGFBP-2 ELISA kit from RayBiotech, according to the manufacturer's protocol.

### Statistical analysis

Results are expressed as means±S.D. Differences between groups were detected by one-way analysis of variance or a paired Student's *t* test. Differences were considered statistically significant at *P*<0.05.

## RESULTS

### Gene expression profiling in fasting compared with refeeding conditions

**Figure 1:**
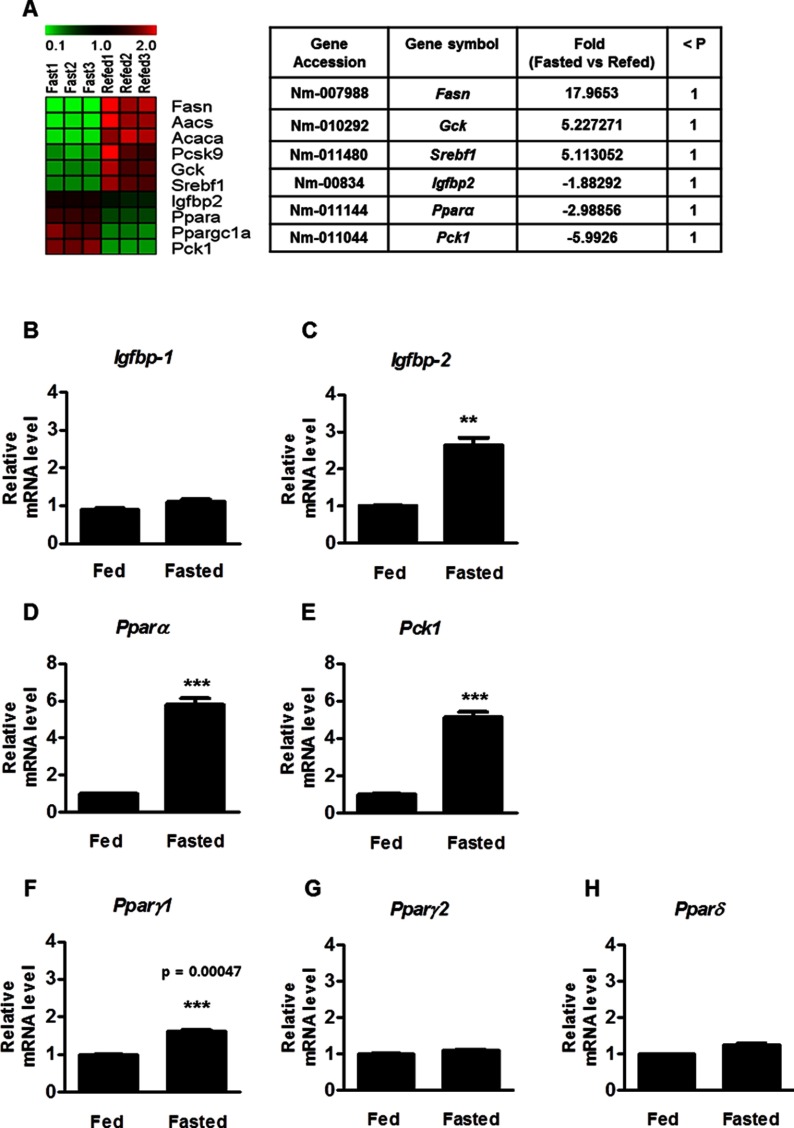
Microarray analysis to identify *Igfbp-2* gene expression level and qPCR analysis in fasting condition (**A**) Hierarchical gene clustering was generated with the TM4 Microarray Software Suite (MeV) from fasted and refed livers. A heat map showing significant changes in a group of selected genes from the livers of mice that were fasted for 24 h and refed for 12 h. The ratios of gene profiles are presented as a heat map (left panel) and gene expression pattern (right panel). Fold change and *P*-values of the genes in the clusters are given on the right. (**B**–**E**) mRNA levels of *Igfbp-1*, *Igfbp-*2, *Pparα* and *Pck1* in the liver of WT mice were measured by qPCR under the indicated conditions. **P<0.01 and ***P<0.001 compared with fed mice.

To uncover the physiological relevance in the livers of fasted and refed WT mice, we first evaluated general gene expression profiles under different nutritional conditions. Previously, high-throughput screening analysis in fasting and refed mouse livers [23] had revealed that *Igfbp*-2 gene expression was higher in the liver of fasting mice than refed mice liver (Figure 1A). *Pparα*, *Pck1* and *Igfbp*-2 mRNA levels were up-regulated in the fasting mice, whereas *Fasn* (fatty acid synthase), *Gck* (Glucokinase) and *Srebf-1* (sterol regulatory element binding transcription factor-1) mRNA were increased after refeeding.

To further evaluate the microarray data, we assessed qPCR analysis in the livers of fed and fasted WT mice. As expected, mRNA levels of *Igfbp-2*, *Pparα* and *Pck1* were increased significantly by the fasting state, relative to the fed state (Figures 1B–1E). In contrast, *Igfbp-1* gene expression was not observed in fasted WT mice. Expression of other PPAR isoforms, such as *Pparγ1*, *PPARγ2* and *PPARδ*, did not change during fasting (Figures 1F–1H). Overall, these findings demonstrated that *Igfbp-2* was up-regulated by the fasting condition.

### Induction of IGFBP-2 by fasting and Wy14643 is dependent on PPARα

**Figure 2:**
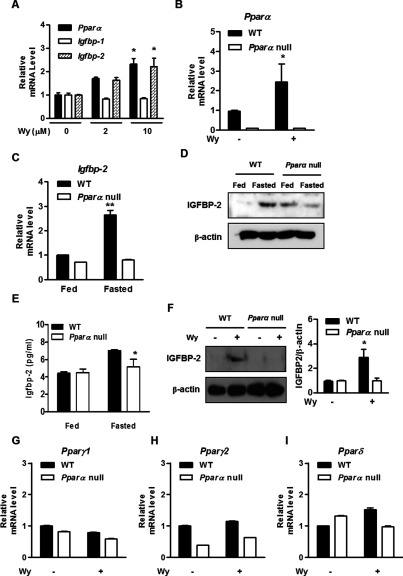
PPARα is involved in the induction of IGFBP-2 following the fasting state and Wy14643 treatment (**A**) Mouse primary hepatocytes were treated with Wy14643 (Wy) for 6 h at the indicated concentrations. Total RNA was isolated and analysed using qPCR with the observed primers. (**B**) Mouse primary hepatocytes from WT and *Pparα* null mice were treated with or without Wy14643 for 6 h. The level of *Pparα* mRNA was measured by qPCR analysis. (**C**) The mRNA level of *Igfbp-*2 in liver from WT and *Pparα* null mice fed and fasted for 24 h was analysed by qPCR. (**D**) Protein level of IGFBP-2 in livers from WT and *Pparα* null mice fed and fasted for 24 h was analysed by Western blot analysis. (**E**) Secretion level of IGFBP-2 *in vivo* and *in vitro*. Mice were fed or fasted for 24 h and serum was collected from WT and *Pparα* null mice. Secretion levels of IGFBP-2 in feeding or fasting conditions were measured by ELISA. (**F**) WT and *Pparα* null mice were treated with or without Wy14643 for 6 h. Whole cell extracts were isolated from primary hepatocytes of the observed conditions and assessed by Western blot analysis with the indicated antibody. (**G**–**I**) Primary hepatocytes from WT and *Pparα* null mice were treated with or without Wy14643 for 6 h. The level of *Pparγ1*, *PPARγ2* and *PPARδ* mRNA was measured by qPCR analysis. *P<0.05 and **P<0.01 compared with untreated control or fed WT mice.

Next, we examined whether PPARα plays a role in regulating *Igfbp-2* gene expression in primary cultured hepatocytes. Wy14643 treatment in hepatocytes resulted in a significant increase in the mRNA levels of *Igfbp-2* and *Pparα* in a dose-dependent manner, but did not affect *Igfbp-1* (Figure 2A). In addition, Wy14643 treatment increased *Pparα* mRNA levels in the hepatocytes of WT mice but not in *Pparα* null mice (Figure 2B). Moreover, *Igfbp-2* mRNA expression increased in fasting WT mice compared with fed or refed mice, in a fashion similar to that shown in Figure 1. However, this phenomenon was not observed in *Pparα* null mice (Figures 2C and 2D). Next, we measured the level of secreted IGFBP-2 in the serum of WT and *PPAR*α null mice subjected to feeding and fasting. The level of IGFBP-2 in the serum was increased in the fasted WT mice (Figure 2E) but not in the *Ppar*α null mice. This indicates that IGFBP-2 secretion as well as mRNA expression of *Igfbp-2* is also increased by PPARα. In accordance with mRNA level, the protein level of IGFBP-2 increased prominently in Wy14643-treated WT mice. Again, this increase in protein level was not observed in Wy14643-treated *Pparα* null mice (Figure 2F). Other PPAR isoforms were not affected by Wy14643 treatment in the primary hepatocytes (Figures 2G–2I). Collectively, these results indicate that PPARα is a key mediator for up-regulating IGFBP-2 expression in primary cultured hepatocytes.

### PPARα regulates the transcriptional activity of IGFBP-2

**Figure 3:**
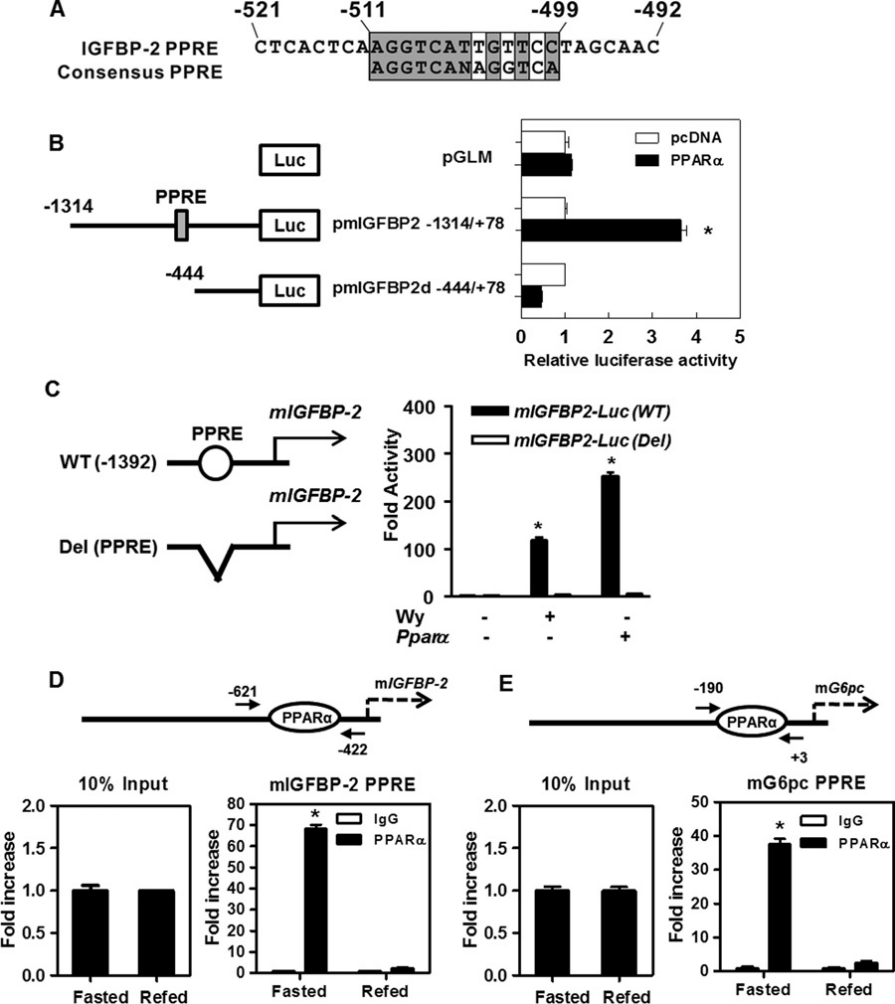
Identification of PPRE in the m*Igfbp-2* promoter (**A**) Comparison of putative PPRE with consensus PPRE sequences. A proposed PPARα-binding element is shaded on the *Igfbp-2* promoter between −511 and −499. The numbers indicate the distance in nucleotides from the transcription start site (+1) of the mouse *Igfbp-2* gene. (**B**) Effects of PPARα on promoter reporter activities in deletion constructs of the *Igfbp-2* gene. Deletion construct of the m*Igfbp-2* promoter was transiently co-transfected with pcDNA3 (open bars) or PPARα expression vector (closed bars) in HEK-293T cells. After 24 h, media were changed to contain 20 μM Wy14643. Luciferase activity was normalized to β-galactosidase activity to correct for transfection efficiency. (**C**) Internal deletion constructs for the *Igfbp-2* promoter were analysed for promoter activity. (**D** and **E**) ChIP assay. Mice were fasted for 24 h and refed for 12 h. Chromatins were isolated from mice livers and ChIP assay was performed. Input represents 10% of purified DNA in each sample. Nuclear extracts from mice livers were immunoprecipitated with anti-PPARα antibody and purified DNA samples were used to perform qPCR with primers binding to the putative PPRE regions on the m*Igfbp-2* (**D**) and m*G6pc* (**E**) gene promoters. All data are representative of at least three independent experiments. *P<0.05 compared with untreated control.

To identify the fundamental molecular mechanism through which PPARα regulates *Igfbp-2* gene transcription, the mouse *Igfbp-2* (m*Igfbp-2*) gene promoter was transiently transfected into human embryonic kidney (HEK)-293T-cells. As shown in Figure 3(A), computer analysis with consensus PPRE sequence showed a highly conserved putative PPRE on the *Igfbp-2* promoter. A proposed PPARα-binding element is shaded in the *Igfbp-2* promoter between −511 bp and −499 bp. The transcriptional activity of m*Igfbp-2* in response to Wy14643 treatment was highest with a full-length gene promoter and was diminished markedly with deletion up to −444 bp (Figure 3B). Moreover, internal deletion of the putative PPRE between −511 bp and −499 bp from the full-length m*Igfbp-2* resulted in decreased promoter activity (Figure 3C). These data collectively suggest that the putative PPRE is located between the −511 and −499 bp regions of the m*Igfbp-2* gene promoter. We next performed ChIP assays in mouse livers to verify PPARα binding to the m*Igfbp-2* gene promoter at the chromatin level. PPARα occupancy was greater on the m*Igfbp-2* promoter (Figure 3D). A known PPRE region on the *G6pc* gene promoter was used as a positive control in the ChIP assay (Figure 3E). These results demonstrate that *Igfbp-2* gene expression is regulated through the direct binding of PPARα to the *Igfbp-2* promoter.

### IGF-1 signalling is altered by PPARα in primary cultured hepatocytes

**Figure 4:**
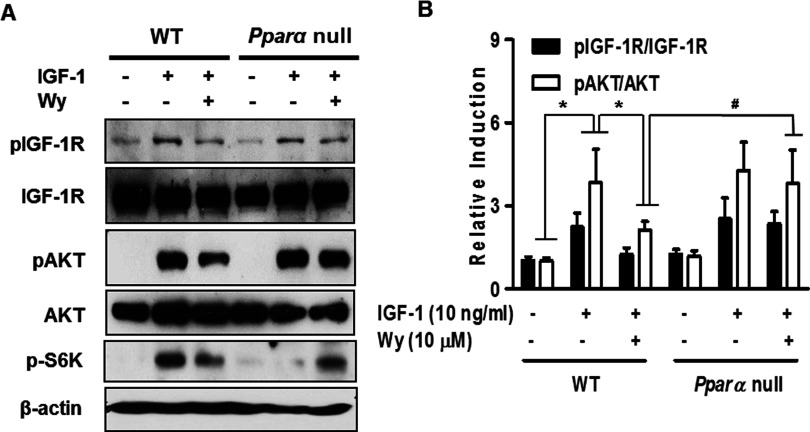
The IGF-1 signalling system is mediated by PPARα in primary hepatocytes WT and *Pparα* null mice were pretreated with Wy14643 for 6 h and then exposed to IGF-1 for 15 min under the indicated conditions. Whole cell extracts were isolated from primary hepatocytes of the indicated groups and assessed by Western blot analysis with various antibodies. Right panel indicate the density of Western blot bands measured with ImageJ software. *P<0.05 compared with untreated control or IGF-1-treated cells and ^#^*P*<0.05 compared with IGF-1- and Wy14643-treated cells.

To determine the role of PPARα on hepatic IGF-1 signalling, we treated hepatocytes of WT and *Pparα* null mice with IGF-1 and/or Wy14643. IGF-1-induced phosphorylation of IGF-1R and Akt were decreased significantly by Wy14643 in WT mice and the inhibitory effect of Wy14643 was abolished in *Pparα* null mice (Figure 4). These findings suggest that PPARα may play a negative role in IGF-1 signal transduction in primary hepatocytes.

### *IGFBP-2* expression is not affected by PPARγ agonist

**Figure 5:**
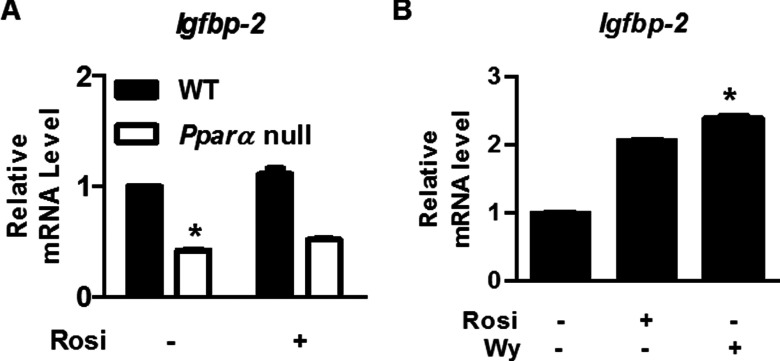
Effects of PPAR agonists in the primary hepatocytes and hepatic cell lines (**A**) Effect of rosiglitazone on *Igfbp-2* gene expression. Rosiglitazone (10 μM) was added to primary hepatocytes for 6 h and RNA was harvested for qPCR. (**B**) mRNA levels of *Igfbp-2* after rosiglitazone or Wy14643 treatment in the HepG2 cell line. Rosiglitazone (10 μM) or Wy14643 (10 μM) was added to HepG2 cells for 6 h and RNA was harvested for qPCR. Values are expressed as mean values±S.E.M. from three experiments. Rosi, rosiglitazone; Wy, Wy14643. *P<0.05 compared with untreated group.

Finally, we asked whether *Igfbp-2* expression was up-regulated by Pparγ agonists. Rosiglitazone, a PPARγ agonist, was added to primary hepatocytes for 6 h and *Igfbp-2* mRNA levels were measured. As shown in Figure 5(A), *Igfbp-2* expression was not up-regulated by rosiglitazone in primary hepatocytes of WT or *Pparα* null mice. Treatment with rosiglitazone in the HepG2 cell line also showed minor effects (Figure 5B). These findings indicate that Pparγ is not involved in *Igfbp-2* expression.

## DISCUSSION

In the present study, we have shown that the fasting state and Wy14643 exposure elevated *Igfbp-2* gene transcription and secretion by promoting PPARα in primary hepatocytes, inhibiting IGF-1 signalling and attenuating an IGF-1-dependent pathway. However, the inhibitory action of Wy14643 on the IGF-1 signalling pathway was disrupted in *Pparα* null mice. Our study showed that Wy14643 exposure increased *Igfbp-2* gene transcription through PPARα recruitment. Based on these findings, we propose that up-regulation of *Pparα* by physiological changes and ligand exposure may prevent hepatic IGF-1 signalling by activating *Igfbp-2* expression.

Changes in IGFBP-2 levels are associated with various physiological and pathological states, such as exercise, pregnancy, aging, hormones, diabetes, obesity, insulin resistance and tumours [24–26]. Previous studies have shown that expression of the *Igfbp-2* gene is promoted significantly in the liver during fasting [27,28] and that insulin markedly attenuates *Igfbp-2* gene expression in myoblast cells and in the heart [29,30]. These findings suggest that IGFBP-2 is altered by physiological states and insulin. However, the biological link between IGFBP-2 and other transcription factors in response to fasting states has not been fully addressed. Our results demonstrate that fasting states elevate IGFBP-2 and PPARα significantly compared with feeding states (Figures 1C and 1D). Remarkably, fasting states and Wy14643 treatment effectively promoted *Igfbp-2* gene expression in WT mice, but not in *Pparα* null mice (Figure 2C). These findings suggest that IGFBP-2 may be involved in fasting conditions and that Wy14643 plays an important role in controlling *Igfbp-2* gene expression in primary hepatocytes.

As mentioned previously, PPARα acts as a key regulator of diverse metabolic processes and also of metabolic homoeostasis [15,16,31]. It has been shown that PPARα reduced cellular functions by attenuating IGF-1R and Akt activity in diverse cancer cells [18–20]. However, there is no evidence of a correlation between PPARα and the IGF-1 signalling system in primary hepatocytes. Our findings reveal a novel action of PPARα involving IGF-1 sensitivity via the IGF-1R–Akt signalling pathway. Our results demonstrate that the stimulation of IGF-1R, Akt, mTOR and S6K activity by IGF-1 exposure is markedly decreased by Wy14643 treatment. The inhibitory effects of Wy14643 were disrupted in PPARα null mice. Thus, PPARα may protect against IGF-1 sensitivity by attenuating the IGF-1-dependent pathway in primary hepatocytes.

It is well known that PPARα regulates the transcription of various target genes [16,21,32,33]. Although our results identify a novel link between PPARα and IGFBP-2 in primary hepatocytes, we do not rule out the possibility that PPARα may rely on other as-yet-unknown mechanisms that control the involvement of transcription co-activators or the completion of co-repressors, protein degradation and modification. Thus, further study is required to determine the detailed molecular network of PPARα and IGFBP-2 in primary hepatocytes.

Ppar isoforms activate target gene expression by binding to the PPRE on the promoter region(s) of target genes [34]. Among the various Ppar isoforms, *Pparα* expression is increased in the liver during fasting, resulting in the stimulation of *Igfbp-2* expression and the secretion of IGFBP-2. In the present study, we demonstrated that *Pparα* was the only PPAR isoform induced in fasted liver (Figures 1 and 5). Additionally, because *PPARγ* expression is very low in the liver during fasting, PPARγ may be not involved in the stimulation of *Igfbp-2* gene transcription. These results indicate that IGFBP-2 is primarily regulated in the liver by PPARα during fasting.

In conclusion, we have shown that the fasting state promotes *Igfbp-2* gene expression by up-regulating PPARα and that the PPARα–IGFBP-2 cascade attenuates IGF-1 sensitivity by controlling the IGF-1 signalling network. Our findings provide evidence of a novel pathway involved in the regulation of the hepatic IGFBP-2–IGF-1 network and/or PPARα in the fasting state as well as a potential therapeutic approach for the treatment of hepatic metabolic disorders.
